# A bidirectional relationship between atrial fibrillation and depression: epidemiology, mechanisms, and clinical implications

**DOI:** 10.3389/fpsyt.2026.1721875

**Published:** 2026-05-28

**Authors:** Wei Luo

**Affiliations:** 1 Department of Cardiology, The First People’s Hospital of Nankang District, Ganzhou, China; 2 Department of Cardiology, Nankang Campus of the First Affiliated Hospital of Gannan Medical University, Ganzhou, China

**Keywords:** atrial fibrillation, clinical implications, depression, epidemiology, mechanisms

## Abstract

Atrial fibrillation (AF) represents a common cardiac arrhythmia that carries substantial morbidity and mortality risks, whereas depression serves as a significant psychological factor affecting cardiovascular health. Recent findings underscore a reciprocal relationship between AF and depression, suggesting that depression may heighten the likelihood of developing AF, while AF may, in turn, worsen depressive symptoms. This review aims to provide a thorough examination of the epidemiological features that underpin this relationship, focusing on population-based research that clarifies prevalence rates and associated risk factors. Furthermore, it delves into the intricate biological and psychosocial mechanisms that connect these two conditions, which include dysregulation of the autonomic nervous system, inflammation, neurohormonal pathways, and behavioral influences. The clinical ramifications of this reciprocal association are also addressed, highlighting the necessity for integrated screening and management approaches to enhance patient outcomes. By consolidating existing research, this article seeks to enrich the understanding of the relationship between AF and depression, as well as to assist clinicians in optimizing therapeutic strategies tailored to address this dual burden.

## 1 Introduction

Atrial fibrillation (AF) is the most common type of clinical arrhythmia, with its prevalence significantly increasing due to global population aging. This disease is closely associated with adverse cardiovascular events such as stroke, heart failure, and increased risk of mortality (1, 2). The pathophysiological mechanisms of AF are diverse, involving factors such as inflammatory signaling, oxidative stress, atrial remodeling, and autonomic nervous system dysregulation (1, 3). Comorbidities such as diabetes, hypertension, chronic kidney disease, and metabolic dysfunction-associated steatotic liver disease have been identified as risk factors for AF, highlighting the complex link between systemic health and atrial arrhythmias (4–6). Additionally, environmental exposures like air pollution and psychosocial stress are increasingly recognized as potential triggers for AF onset (7).

Depression is a prevalent and significant comorbidity among patients with cardiovascular disease (CVD). Epidemiological surveys indicate that approximately 20% of CVD patients exhibit depressive symptoms, with prevalence rates varying by population characteristics and disease type (8, 9). The presence of depression is associated with decreased health-related quality of life (HRQoL), increased symptom burden, and adverse clinical outcomes, including higher rates of hospitalization and mortality (10, 11). Its pathophysiological links involve shared mechanisms such as increased sympathetic nervous system activity, hypothalamic-pituitary-adrenal (HPA) axis dysregulation, systemic inflammation, and endothelial dysfunction (9, 12). Furthermore, psychosocial factors like anxiety, social isolation, and stress further exacerbate this association (13, 14).

In recent years, the bidirectional relationship between AF and depression has garnered increasing attention. Large cohort studies and meta-analyses suggest that depression is associated with an increased risk of developing AF, while the diagnosis of AF and its symptom burden can also induce or worsen depression (15–19). However, epidemiological evidence shows non-negligible heterogeneity in effect size estimates and study designs; research on biological mechanisms faces a translational gap when extrapolating from animal models to human disease; and the evidence base for clinical management recommendations remains insufficient. This review aims to systematically evaluate the epidemiological data in this field (with a focus on study design, confounding control, and generalizability), synthesize mechanistic evidence (clearly distinguishing established pathways from speculative ones), assess the strength and limitations of evidence for clinical implications, and propose stratified practice recommendations and targeted future research directions.

## 2 Main body

### 2.1 Epidemiological characteristics of AF and depression

#### 2.1.1 Prevalence and association of AF and depression

##### 2.1.1.1 Key epidemiological findings

Multiple large-scale population-based studies have reported a positive association between depression and the risk of atrial fibrillation. A nationwide cohort study in South Korea (n > 5 million) reported that the cumulative incidence of newly diagnosed atrial fibrillation during a 10-year follow-up was 4.44% in depressed patients and 1.92% in non-depressed individuals, with multivariate adjustment showing that depression was associated with an approximately 25% increased risk of atrial fibrillation, and the risk was more significant for recurrent depression (16). Meta-analyses synthesizing existing evidence estimated that the pooled hazard ratio (HR) for depression associated with increased risk of atrial fibrillation was approximately 1.15 (95% CI: 1.05–1.25), and the pooled HR for antidepressant use and atrial fibrillation risk was approximately 1.16 (95% CI: 1.08–1.25) (20). Additionally, prospective cohorts such as the ARIC study have also reported associations between anger, exhaustion, antidepressant use, poor social connections, and the occurrence of atrial fibrillation (17).

Among patients with atrial fibrillation, the prevalence of depression is estimated to be 20% to 40%, although reported values vary considerably across studies (18). In large cohorts, prevalence estimates based on diagnostic codes and questionnaire screening fall within a narrow range of 17.8%–19.9% (21, 22). Notably, mental health conditions (MHCs) not only affect anticoagulant therapy but are also associated with lower utilization of rhythm control strategies such as anti-arrhythmia agents and catheter ablation, suggesting that depression may systematically impact the comprehensive treatment of atrial fibrillation (23). In specific populations, such as patients with congenital heart disease complicated by atrial arrhythmias, patient-reported outcomes are more significantly impaired (24). Type D personality is closely associated with decreased quality of life in patients with atrial fibrillation (25). Genetic epidemiological studies using Mendelian randomization further support a causal relationship between depression and atrial fibrillation, and suggest that hypertension and obesity may act as mediating factors (19).

##### 2.1.1.2 Critical appraisal of existing evidence

Despite the overall consistency of these findings, several important methodological issues in the existing literature warrant careful consideration during interpretation.

First, there is heterogeneity in the assessment methods for depression and AF. Definitions of depression vary across studies, ranging from diagnostic codes (ICD codes) and prescription records for antidepressants to self-report scales (e.g., PHQ-9, CES-D). Differences in case ascertainment criteria can introduce misclassification bias and lead to variability in prevalence estimates and effect sizes. The detection of AF also varies; studies based on administrative databases may miss cases of asymptomatic or paroxysmal AF.

Second, residual confounding is a significant concern. Depressed individuals typically exhibit more adverse health behaviors (e.g., physical inactivity, smoking, poorer diet) and a higher burden of cardiovascular comorbidities. Although most studies attempt statistical adjustment for these factors, unmeasured or imprecisely measured confounders may still overestimate the independent effect of depression. Only a few studies have employed sibling comparison designs or instrumental variable methods to further control for unmeasured family-level and environmental confounding.

Third, regarding generalizability, the vast majority of large-scale epidemiological studies originate from high-income countries (e.g., South Korea, Nordic countries, USA, UK), with a lack of high-quality data from low- and middle-income regions (South Asia, Africa, Latin America). The few available studies from low- and middle-income countries (e.g., the Libyan INSPECT study (8) and a post-COVID-19 survey in China (26)) suggest that prevalence rates and risk factor profiles may differ from those in high-income countries, but the evidence remains very limited. Given the significant differences in cardiovascular risk profiles, mental health service accessibility, and sociocultural factors in these regions, the extrapolation of current conclusions is limited.

Finally, most studies are prospective cohort designs, which cannot completely rule out the possibility of reverse causation. Depression could be an early manifestation (i.e., prodromal symptom) of undiagnosed AF or other physical illnesses, a possibility often inadequately considered in design and analysis. Sex-stratified analyses show gender differences in the distribution of sleep and psychological distress among cardiovascular patients (14), highlighting the need to consider subgroup-specific effects.

##### 2.1.1.3 Summary

Epidemiological evidence generally supports an independent association between depression and an increased risk of developing AF. However, the effect sizes are relatively modest (HR ~1.15–1.25) and may be influenced by varying degrees of residual confounding. The high prevalence of depression in AF patients (approximately 20–40%) is consistently reported, but precise estimates depend on assessment tools and population characteristics. Future research should employ standardized prospective assessment tools, prioritize the inclusion of representative populations from low- and middle-income regions, and utilize quasi-experimental designs (e.g., instrumental variables, negative controls) to strengthen causal inference (see **Table 1**).

**Table 1:** Summary of major epidemiological studies on the association between atrial fibrillation and depression.

Research (First Author, Year)	Research Type	Sample Size/Population	Exposure/Outcome Assessment Methods	Main Effect Estimation (95% CI)	Limitation
Kim YG, 2022 (16)	National Retrospective Cohort	Korean Adults>5 Million	Depression: ICD-10 code; Atrial fibrillation: diagnostic code	Depression vs. non depressive new onset atrial fibrillation aHR 1.251 (higher risk of recurrent depression)	Relying solely on administrative data; There may be unmeasured confounding; Single race population
Fu Y, 2022 (20)	Systematic Review/Meta-analysis	Multiple prospective cohorts with inconsistent total sample size	Depression: Scale/Diagnosis; Antidepressants: prescription records	Depression HR 1.15; Antidepressant HR 1.16	The original research has significant heterogeneity; Some studies have not adequately adjusted for lifestyle factors
Jaakkola J, 2022 (21)	National Prospective Cohort	Atrial fibrillation patients in Finland	Mental Health Conditions (MHC): Diagnostic Code; Oral anticoagulant use	The prevalence of MHC is approximately 17.8% -19.9%; MHC is associated with lower anticoagulation initiation rate	The focus is on therapeutic use and non pathogenic association; MHC contains multiple diagnoses
Teppo K, 2022 (22)	National Prospective Cohort	Newly diagnosed atrial fibrillation patients in Finland	MHC: Diagnostic Code; DOAC sustainability	MHC is associated with increased risk of non sustained use of DOAC	Only prescription data is available, unable to distinguish between intentional discontinuation and loss to follow-up
Zhou H, 2024 (19)	Mendelian Randomization	Publicly disclose GWAS summary data (mainly of European descent)	Genetic instrumental variables represent depression, anxiety, and atrial fibrillation	Depression has a causal effect on atrial fibrillation; Hypertension and obesity act as mediators	Mainly based on the European population; The MR hypothesis may be influenced by horizontal pleiotropy
Garg PK, 2021 (17)	Prospective Cohort	Approximately 13000 community members in the United States	Anger, exhaustion, use of antidepressants, social connections; Atrial fibrillation event	The use of antidepressants increases the risk of atrial fibrillation; Exhaustion has an associated trend	Exposure is a single assessment; Possible residual mixing
Polikandrioti M, 2021 (18)	Narrative Review	Atrial fibrillation patient	Depression/Anxiety Assessment	The prevalence of depression in patients with atrial fibrillation is 20% -40%	Non systematic, with significant variations in evidence strength
Lapa ME, 2023 (27)	Prospective Cohort	Patients with atrial fibrillation using DOACs	Depression diagnosis; DOAC compliance (percentage of coverage days)	Depression reduces the OR of full compliance by approximately 0.88-0.89	Based solely on claim data; Unable to assess medication motivation
Teppo K, 2022 (23)	National Cohort	Atrial fibrillation patient	MHC; Rhythm control strategies (antiarrhythmic drugs, ablation)	MHC is associated with lower utilization of rhythm control	Only evaluate treatment options, without directly assessing recurrence

#### 2.1.2 Impact of depression on AF patients

##### 2.1.2.1 Quality of life and functional status

The presence of depression in AF patients is associated with significantly lower health-related quality of life (HRQoL). Assessed by tools such as SF-36 and AFEQT, AF patients with comorbid depression report lower physical and mental health scores, along with greater perceived symptom severity and functional limitations (18, 28, 29). Some longitudinal studies suggest that long-term improvement in depressive symptoms after radiofrequency catheter ablation is associated with subsequent improvements in HRQoL (28), but most evidence is cross-sectional, and the direction of causality requires further validation. Cluster analysis of cognitive and behavioral responses in AF patients also reveals different psychological adaptation patterns, further influencing quality of life (29).

##### 2.1.2.2 Adverse clinical outcomes

Observational studies consistently report that AF patients with comorbid depression face higher rates of all-cause and cardiovascular hospitalizations (15). Regarding mortality, the direction of evidence is consistent, but the magnitude of the effect varies across studies, and isolating the independent effect of depression from the confounding effects of disease severity and comorbidity burden remains challenging. Depression may also indirectly affect prognosis by reducing treatment adherence (particularly to anticoagulation and rhythm control) (see Section 2.3). Psychosocial risk factors such as low health literacy and insufficient social support further undermine patient treatment adherence (30).

##### 2.1.2.3 Cognitive function and frailty

Emerging evidence suggests that depression in AF patients is associated with cognitive decline and increased frailty indices (31–34). Systematic reviews and meta-analyses indicate that depression and cardiovascular risk factors have a synergistic effect on cognitive impairment in AF patients (34), suggesting that mental, neurological, and physical functions may form a synergistic pattern of multimorbidity in AF patients. However, research in this area is currently limited, mostly cross-sectional or with short follow-up periods, requiring further validation in prospective studies.

##### 2.1.2.4 Evidence assessment

Major limitations in this area include: depression assessment in most studies is based on single screening rather than clinical diagnostic gold standards; assessment of HRQoL and symptoms may be confounded by reverse causation (i.e., those with more severe symptoms are more prone to depression); interventional evidence is relatively scarce, with evidence for the effect of psychological interventions on patient-centered outcomes mainly coming from small trials and pre-post studies. Notably, there is a significant gap between physicians’ and patients’ awareness, perception, and attitudes towards depressive states in AF, leading to under-recognition and under-treatment of depression in this population (35).

##### 2.1.2.5 Summary

Existing evidence strongly suggests that depression is an important independent correlate of reduced HRQoL and increased hospitalization rates in AF patients. However, the evidence strength for the independent effects of depression on mortality, cognitive decline, and frailty is moderate, requiring validation by more high-quality longitudinal studies. In clinical practice, routine assessment of depressive status in AF patients should be considered a fundamental element of quality care.

### 2.2 Biological mechanisms linking AF and depression

The mechanistic link between AF and depression involves multiple levels. This section categorizes mechanisms into two groups based on the maturity of evidence: (1) mechanisms established by human studies and replicated evidence from independent laboratories; (2) speculative mechanisms primarily based on animal models, awaiting human validation.

#### 2.2.1 Established mechanistic pathways

##### 2.2.1.1 Autonomic nervous system dysfunction

Autonomic imbalance is one of the most consistently identified mechanistic links between AF and depression. Human studies have well established that depressed patients exhibit increased sympathetic activity and decreased parasympathetic tone, objectively indicated by reduced heart rate variability (HRV) (36). Reduced HRV is a recognized marker of poor autonomic regulation and is associated with an increased risk of AF onset and recurrence. Elevated resting heart rate and delayed heart rate recovery observed in depressed patients further support the presence of chronic sympathetic hyperactivation. Autonomic imbalance contributes to the initiation and maintenance of AF by promoting atrial electrical remodeling (increased dispersion of refractoriness) and structural substrate transformation (fibrosis) (37). It is worth emphasizing that autonomic dysfunction is a well-validated predictor of AF recurrence in patients and is a clinically modifiable factor through pharmacotherapy (e.g., beta-blockers) and lifestyle interventions. The psychological aspects of AF involve multiple dimensions including onset, cognition, prognosis, and symptom perception (37). Mathematical modeling studies also provide a quantitative framework for understanding the electrophysiology of atrial myocytes and cardiac mechanics integration (38).

##### 2.2.1.2 Systemic inflammation

Evidence from population studies and clinical samples consistently indicates that depression is associated with a state of low-grade chronic inflammation, manifested by elevated circulating levels of C-reactive protein (CRP), interleukin-6 (IL-6), and tumor necrosis factor-alpha (TNF-α) (39–42). On the other hand, the role of inflammation in atrial electrical and structural remodeling is well established (43–45). The atrial inflammatory characteristics of different AF subtypes and their relationship with clinical risk factors have been studied (43). Prospective studies show that elevated inflammatory markers can predict AF onset and recurrence after ablation (46). Mechanisms by which inflammation promotes AF include inducing atrial fibrosis, altering ion channel function, and impairing connexin protein expression. Specifically, TNF-α can participate in the regulation of IKur in atrial myocytes via activation of PKCα (39). The systemic immune-inflammation index (SII) has been shown to independently predict the presence of left atrial thrombus in patients with non-valvular AF (47). In elderly patients with persistent AF, an association exists between frailty and inflammation (48). Therefore, inflammation constitutes a core established pathway linking depression and AF. However, human longitudinal mediation analyses directly proving that depression-induced inflammation leads to AF remain scarce, representing a critical evidence gap in current mechanistic research.

#### 2.2.2 Speculative pathways supported by preliminary evidence

##### 2.2.2.1 P2X7 receptor/NLRP3 inflammasome pathway

Animal experiments provide direct evidence linking P2X7 receptor (P2X7R) activation via the NLRP3 inflammasome pathway to depressive-like states and AF susceptibility. In chronic unpredictable stress (CUS) rodent models, CUS leads to upregulation of P2X7R and NLRP3 inflammasome components, accompanied by increased markers of sympathetic sprouting (TH, GAP43) and abnormal expression of ion channel proteins (Nav1.5, Cav1.2, Kv4.3, etc.), ultimately increasing AF inducibility. Pharmacological inhibition or genetic knockout of P2X7R attenuates atrial fibrosis, restores ion channel expression, and reduces AF susceptibility (36). Although this pathway is mechanistically clear and consistent with the inflammatory hypothesis of human depression, clinical trial data for P2X7R inhibitors in human AF prevention are currently lacking, and its translatability to human disease remains to be confirmed.

##### 2.2.2.2 IL-6 trans-signaling

Selective IL-6 trans-signaling has been proposed as an important effector pathway for atrial remodeling. Animal models show that blocking IL-6 trans-signaling attenuates atrial fibrosis, conduction abnormalities, and oxidative stress, reducing AF inducibility (3). Although elevated IL-6 levels are a common feature of human depression and AF, the effects of pathway-specific interventions mentioned above have not been validated in humans.

##### 2.2.2.3 Oxidative stress

Depression may promote atrial structural remodeling by enhancing oxidative stress. In rodent models, increased markers of oxidative stress are associated with the activation of profibrotic pathways (e.g., ROS/p-p38MAPK). Pinocembrin has been shown to reduce AF susceptibility in isoproterenol-induced rat models (49) and to decrease AF susceptibility in rodent depression models (50). Dapansutrile (NLRP3 inhibitor) improves atrial inflammation and AF susceptibility in HFpEF rats (51). However, high-quality evidence directly linking peripheral oxidative stress markers to atrial substrate changes and clinical AF outcomes in human studies remains limited.

##### 2.2.2.4 Limitations of extrapolation from animal models to humans

When interpreting the above animal experimental evidence, it is crucial to consider the following key limitations: Animal depression models (e.g., CUS, lipopolysaccharide-induced) only partially mimic the chronic, recurrent, and psychosocial complexity of human depression; the environment for atrial electrophysiology (*in vivo* neurohumoral regulation vs. ex vivo heart preparations) significantly affects arrhythmia inducibility; species differences exist in injury-protection pathways, and the pharmacological properties of P2X7R antagonists differ between rodents and humans. Therefore, these pathways should currently be considered speculative mechanisms awaiting human validation, rather than established targets ready for clinical intervention.

##### 2.2.2.5 Summary

**Figure 1:**
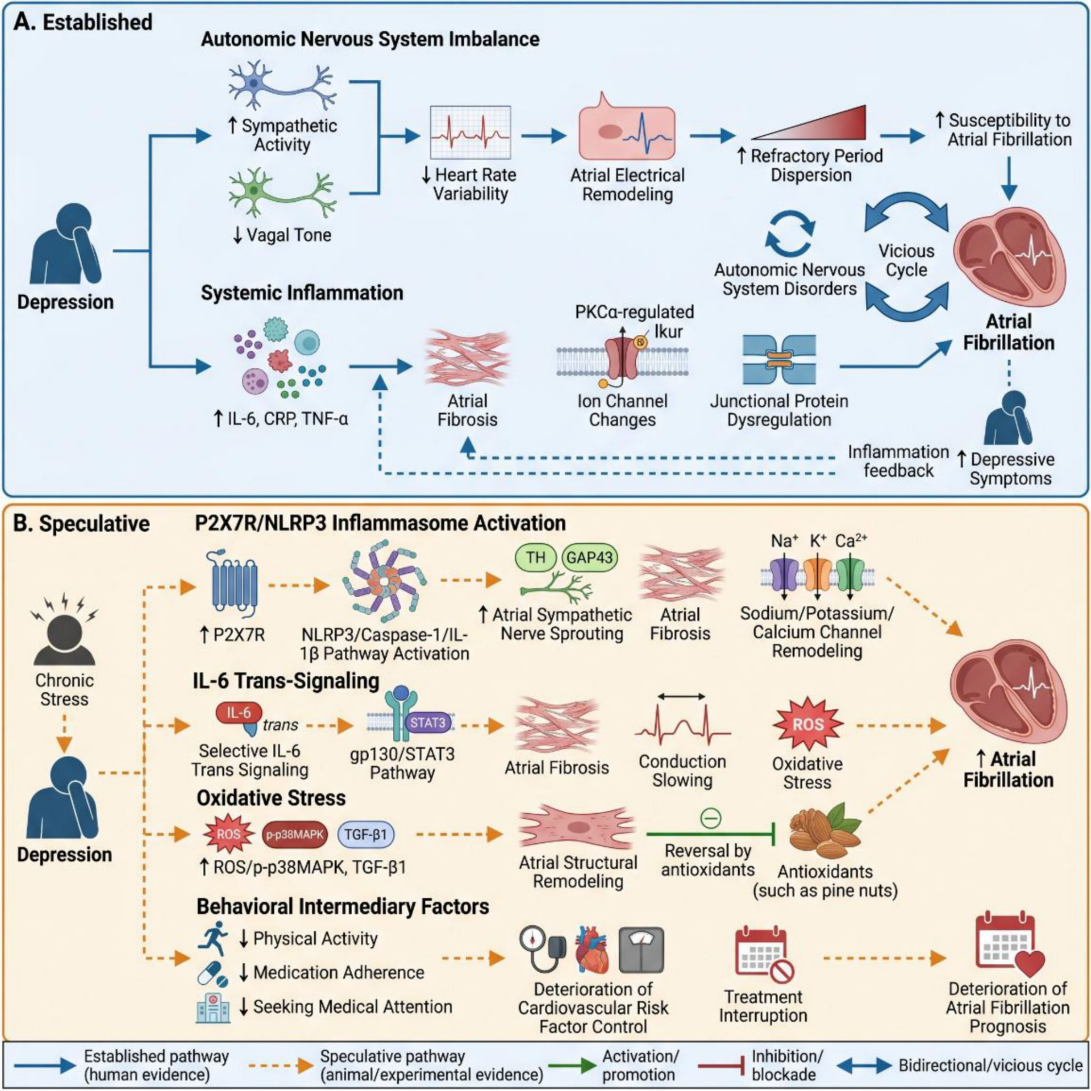
Mechanism pathway framework diagram of the bidirectional relationship between AF and depression.

Multiple shared mechanistic pathways are involved between AF and depression. Autonomic nervous system dysfunction and systemic inflammation are the mechanisms with the most robust evidence, repeatedly validated in human studies. The P2X7R/NLRP3 pathway and IL-6 trans-signaling show potential as therapeutic targets in animal models, but their translational significance for human disease awaits prospective clinical studies. Future mechanistic research should prioritize human mediation analyses and interventional experiments to move from association to causation (see **Figure 1**).

### 2.3 Impact of depression on treatment outcomes in atrial fibrillation

#### 2.3.1 Anticoagulation therapy adherence

##### 2.3.1.1 Summary of evidence

Anticoagulation therapy is the cornerstone of stroke prevention in AF, but poor adherence is a prominent clinical issue in AF patients with comorbid depression. Studies show that compared with non-depressed patients, depressed patients have an approximately 11–12% lower probability of adequate and optimal adherence to direct oral anticoagulants (DOACs) (27). Notably, this adherence difference has not been consistently observed with warfarin use, possibly because the therapeutic monitoring model of warfarin (regular INR testing) provides patients with regular contact with the healthcare system, thereby partially offsetting the negative impact of depression on adherence. Longitudinal studies indicate that oral anticoagulant adherence trajectories follow different patterns, including sustained adherence, gradual decline, and early discontinuation (52). For patients switching from warfarin to DOACs, their previous time in therapeutic range (TTR) is significantly associated with subsequent DOAC adherence (53).

##### 2.3.1.2 Determinants and complexity

Anticoagulation adherence is influenced by multi-level factors. At the medication level, once-daily DOAC regimens (e.g., edoxaban) show higher adherence and persistence rates compared with twice-daily regimens and vitamin K antagonists (54); increased copayment is significantly associated with higher discontinuation rates (55). Label non-adherence (especially underdosing) is common and associated with adverse outcomes (56, 57). At the patient level, the impact of mental health conditions (MHCs) on adherence has been extensively studied. Beyond depression (58), the effect of anxiety disorders on anticoagulation adherence indicates that anxiety does not significantly reduce adherence but may affect other aspects of treatment engagement (59). Medication-related concerns are an independent predictor of non-adherence, with predictive value independent of depressive symptoms themselves (60). This suggests that clinical interventions need to target both depressed mood and medication beliefs as modifiable factors. Approximately 25% of patients discontinue anticoagulation within one year; younger age, fewer comorbidities, and low self-efficacy are additional risk factors for non-adherence (61, 62). Anticoagulation non-adherence is closely associated with a significantly increased risk of ischemic stroke and systemic embolism (63). Of particular concern, hospitalization for thromboembolic events does not automatically improve post-discharge adherence (64), indicating that experiencing an adverse event cannot replace structured adherence interventions. Predictive factors for adherence to recommended anticoagulation after discharge from a stroke unit have been studied, with education level and clarity of the discharge medication regimen being key factors (65).

##### 2.3.1.3 Intervention evidence and clinical recommendations

Multiple interventions show potential for improving adherence. Nurse-led behavioral activation programs and shared decision-making frameworks enhance patient empowerment and treatment satisfaction (66–68). These interventions have been validated by randomized controlled trials and are now included in the European Society of Cardiology guidelines for AF management. Based on existing evidence, the following stratified recommendations are proposed for anticoagulation management in AF patients with comorbid depression:

Strong Recommendations (Supported by Higher-Level Evidence)

1. Routine screening for depression in AF patients before initiating anticoagulation, with regular follow-up reassessments;
2. Use of a shared decision-making framework to actively assess and address patients’ concerns and beliefs about anticoagulation;
3. Preference for once-daily DOAC regimens to improve adherence.

Reasonable Recommendations (Supported by Moderate-Level Evidence, Requiring Clinical Judgment)

1. Consider initiating nurse-led adherence support programs for patients with comorbid depression;
2. Provide structured anticoagulation education during the discharge transition period, clearly specifying drug name, dose, and administration method, ensuring patient understanding.

##### 2.3.1.4 Areas for further research

Direct comparisons of the additional benefits of psychological interventions (e.g., CBT, behavioral activation) on improving anticoagulation adherence are lacking, with no randomized controlled trials using cardiovascular outcomes as the primary endpoint.

#### 2.3.2 Recurrence after catheter ablation

##### 2.3.2.1 Clinical background

Catheter ablation is an effective rhythm control strategy for symptomatic AF, but post-procedural recurrence rates remain high, especially in populations with comorbidities. Left atrial enlargement, atrial fibrosis, heart failure, coronary artery disease, and autonomic dysfunction are known predictors of recurrence (69–71). Additionally, in patients with persistent AF, sex differences influence atrial remodeling and post-ablation recurrence (69), and post-ablation left atrial function affects long-term recurrence rates (70).

##### 2.3.2.2 Evidence linking depression and ablation recurrence

Observational studies and meta-analyses suggest that AF patients with depression after ablation face a higher risk of recurrence (some studies report approximately a two-fold increased risk) (72). Depression may increase recurrence risk through multiple pathways: autonomic imbalance (slower heart rate recovery is an indicator of autonomic dysfunction and has been confirmed to be associated with post-ablation recurrence (73)); chronic inflammation (a pro-inflammatory state promotes re-arrhythmogenic remodeling of the atrial substrate after ablation, and elevated hs-CRP is a predictor of early recurrence (46)); and behavioral factors (depression affects adherence to post-procedural medication and lifestyle recommendations (74)). The prevalence of psychological distress among AF ablation patients is noteworthy, with depression and anxiety symptoms being quite common at the time of cardioversion or ablation (75). Sex differences also exist in outcomes after persistent AF ablation (76). Furthermore, patients with comorbidities such as chronic obstructive pulmonary disease (77) and asthma (78) have significantly higher rates of AF recurrence after ablation, and these conditions share inflammatory and autonomic pathways with depression.

##### 2.3.2.3 Limitations of the evidence

It must be clearly stated that the number of causal studies specifically assessing the relationship between depression and post-ablation recurrence is limited. Existing evidence primarily comes from:

*Post-hoc* analyses not primarily focused on mental health exposures, which may not have systematically assessed depression;

Indirect inferences from mechanisms such as autonomic, inflammatory, and adherence pathways; prospective interventional trials directly demonstrating that antidepressant or psychological interventions can reduce post-ablation recurrence rates are absent. Currently, only studies evaluating the effect of ablation on psychological distress exist, such as a randomized clinical trial comparing the impact of AF catheter ablation versus drug therapy on psychological distress (79).

Most studies do not differentiate between baseline depression and new-onset post-ablation depression (the latter occurring in approximately 17% of cases and associated with factors such as younger age and sedation level (80)), and the prognostic significance of these two conditions may differ. From a procedural perspective, the effects of repeat *in-situ* ablation versus extensive ablation in recurrent persistent AF differ (81), and the extended outcomes of left atrial posterior wall isolation have also been evaluated in randomized trials (82).

##### 2.3.2.4 Clinical recommendations (reasonable recommendations based on current evidence)

Given the biological plausibility and preliminary clinical evidence that depression affects ablation outcomes, the following recommendations can be made:

For AF patients scheduled for elective catheter ablation, systematic screening for depression and anxiety should be performed preoperatively. A cross-sectional survey showed that anxiety and depression are quite common among AF patients undergoing catheter ablation, influenced by factors such as disease duration and socioeconomic status (83);

Patients with moderate-to-severe depressive symptoms should be considered for referral to mental health professionals for evaluation and intervention before ablation;

Patients at high risk for post-ablation depression (younger age, preoperative depression, high anxiety levels) should be included in regular mental health follow-up plans. Early identification and intervention may improve ablation outcomes.

##### 2.3.2.5 Research gap

There is an urgent need for large-scale randomized controlled trials in patients awaiting catheter ablation to evaluate whether integrated psychological interventions (e.g., perioperative CBT) can reduce arrhythmia recurrence rates and improve quality of life.

### 2.4 Interaction between atrial fibrillation and depression: clinical management challenges and responses

#### 2.4.1 Clinical dynamics of bidirectional influence

The relationship between AF and depression constitutes a self-reinforcing vicious cycle. Symptoms of AF—palpitations, fatigue, dyspnea, decreased exercise tolerance—can trigger fear, helplessness, and activity avoidance, promoting or worsening depression. Anticipatory anxiety about the unpredictability of AF episodes can lead to social isolation and further decline in quality of life. On the other hand, depressed patients are more likely to neglect health behaviors, have reduced adherence to medications and follow-up, and directly or indirectly increase AF burden through biological pathways such as autonomic imbalance and inflammation (15, 18). Understanding this mutually reinforcing relationship is the logical starting point for designing effective interventions: breaking the vicious cycle requires both optimizing cardiac treatment and systematically managing mental health.

Genetic evidence (Mendelian randomization) supports a causal effect of depression on AF and suggests that hypertension and obesity are important mediating factors (19). This finding has clinical value—optimizing blood pressure control and weight management in AF patients with comorbid depression may partially mitigate biological risk. The association between obesity and cardiovascular disease, along with advances in pharmacotherapy, further supports the importance of weight management in primary and secondary prevention of AF (84). The pathophysiology of hypertensive heart failure also provides context for understanding the impact of pressure overload on the heart (85). However, the effectiveness of these interventions requires validation in prospective trials.

#### 2.4.2 Existing evidence for integrated management and stratified clinical recommendations

Integrating mental health management into standard AF care is gaining conceptual acceptance, but its implementation in practice remains insufficient.

##### 2.4.2.1 Strength of evidence for existing interventions

Cognitive Behavioral Therapy (CBT): In patients with arrhythmias, small RCTs and pre-post studies show that CBT can reduce anxiety and depression scores and improve the psychological dimension of HRQoL (86, 87). A Cochrane systematic review and meta-analysis focusing on patients with coronary heart disease, heart failure, or AF provides higher-level evidence for the effectiveness of CBT (87). However, most studies have small sample sizes, short follow-up periods (often 3–6 months), and do not use cardiovascular events as the primary endpoint. A preliminary randomized controlled trial using a SMART approach to reduce symptoms of paroxysmal AF demonstrates the feasibility of innovative trial designs (88).

Nurse-Led Integrated Management: Nurse-led comprehensive AF management programs have been shown in randomized trials to reduce cardiovascular hospitalizations and cardiovascular mortality and improve psychological outcome measures (89). Adding targeted psychological interventions (e.g., behavioral activation) to this model shows additional benefits in improving depression and quality of life (90), making it the most clinically feasible and evidence-supported integration strategy. The impact of early rehabilitation nursing on post-procedural cardiac function and quality of life in AF patients further supports the key role of nurses in comprehensive management (90).

Digital Health and Patient-Reported Outcome Measures (PROMs): Digital platforms used to track patients’ psychological status and symptoms have improved adherence and quality of life in some studies. A randomized clinical trial on the effectiveness of digital animation-based multi-stage education for AF patients undergoing catheter ablation (91) and post-ablation digital monitoring with electronic patient-reported outcome collection (92) both show promise for digital health technologies, but the degree of benefit and cost-effectiveness require further evaluation.

Family-Centered Interventions: The improvement of clinical outcomes in AF patients through short-term family-centered interventions has been evaluated in a randomized clinical trial (93), highlighting the potential value of involving families in care plans.

##### 2.4.2.2 Stratified clinical practice recommendations

Based on current evidence, the following stratified recommendations are proposed to guide clinical management (see **Table 2** for details). Integrated management strategies primarily include: routine screening for depression and anxiety (PHQ-9/GAD-7) in AF patients; implementing nurse-led comprehensive management incorporating mental health support; optimizing anticoagulation adherence through shared decision-making and prioritizing once-daily DOAC regimens; considering CBT or behavioral activation for patients with mild-to-moderate depression; conducting psychological assessment before ablation and regular monitoring post-ablation; and using digital health tools to track psychological status. Meanwhile, the impact of psychological interventions on hard cardiovascular endpoints, the net benefit of conventional antidepressant medications on AF outcomes, and the effectiveness of family-caregiver psychological interventions require further research validation. The role of early risk factor modification and ablation in preventing atrial substrate remodeling is also considered potentially important (96).

**Table 2:** Integrated management strategies for patients with atrial fibrillation and depression: evidence strength and stratified clinical recommendations.

Level of Evidence	Strategy	Key supporting literature	Specific suggestions
Higher (should be implemented)	Routine depression/anxiety screening	(18, 21, 22, 31, 32)	Use PHQ-9/GAD-7 screening at least once a year when diagnosing atrial fibrillation or making significant adjustments to treatment.
	Nurse led comprehensive management + psychological health support	(66, 68, 86, 89, 90)	Incorporate mental health assessment into the standard atrial fibrillation nursing pathway; Nurses act as coordinators.
	Shared decision-making improves anticoagulant compliance	(27, 54, 60, 67)	Proactively discuss patient concerns when initiating anticoagulation; Prioritize choosing DOAC once a day.
Moderate (can be considered)	Cognitive Behavioral Therapy (CBT) / Behavioral Activation	(68, 86, 87)	Provide CBT or behavioral activation on the basis of standard care for patients with mild to moderate depression.
	Psychological assessment before ablation and postoperative monitoring	(72, 75, 80, 83)	Preoperative screening for depression; Follow up psychological status at 1, 3, and 6 months after surgery.
	Digital Health + Patient Reported Outcome Tracking	(91, 92)	Applications or platforms track psychological symptoms, automatically alert and push educational content.
Need to study (weak evidence)	The impact of psychological intervention on cardiovascular hard endpoints	(79) (only involving the psychological impact of ablation)	RCTs are needed to evaluate the combined psychological cardiac intervention with stroke and death as endpoints.
	Net benefit of atrial fibrillation outcomes with conventional antidepressant drugs	(20, 94)	Pharmacoepidemiological studies evaluate the impact of SSRI/SNRI on atrial fibrillation burden and bleeding risk.
	Psychological intervention for family caregivers	(93, 95)	Can family centered interventions indirectly improve patient prognosis.

##### 2.4.2.3 Mental health of caregivers

Notably, the prevalence of anxiety and depression is also high among caregivers of AF patients, influenced by factors such as the severity of the patient’s condition and caregiving burden (95), which may indirectly affect the quality of support patients receive and their health outcomes. Assessing caregiver stress and providing them with appropriate support should be part of patient-family-centered comprehensive care.

##### 2.4.2.4 Screening and management pathway for depression in AF patients

**Figure 2:**
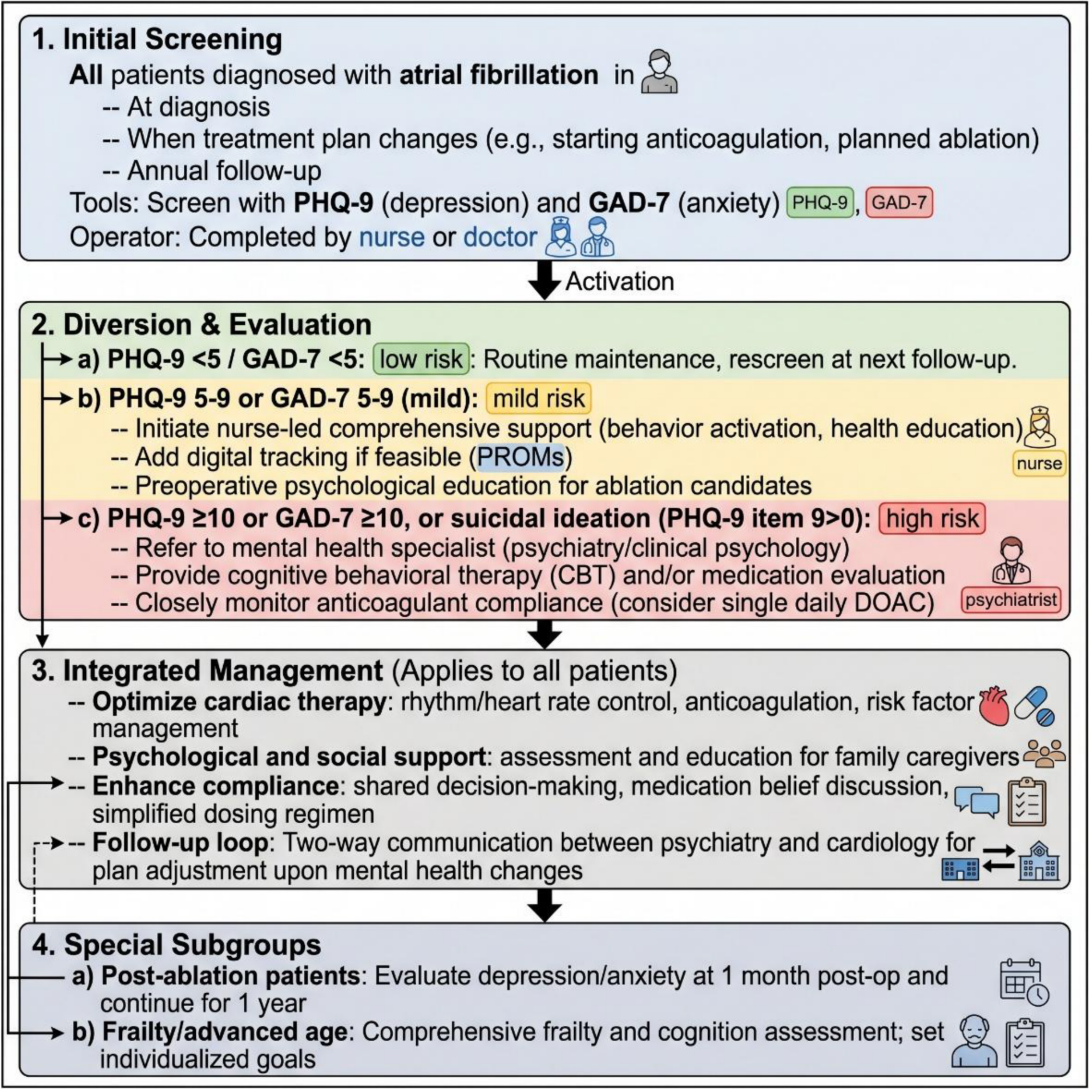
Recommendations for the screening, evaluation, and management process of depression in patients with AF.

This article further proposes an integrated clinical pathway for the screening, assessment, and management of depression in AF patients. The pathway starts with an initial screening node, recommending systematic screening using PHQ-9 and GAD-7 for all patients with confirmed AF at the initial assessment, at the time of treatment changes, and during annual follow-up. Based on the scores, patients are stratified into three levels: routine maintenance; initiation of nurse-led comprehensive support and digital tracking for mild symptoms; and referral to mental health specialists for CBT or medication evaluation for moderate-to-severe symptoms or suicidal ideation. Regardless of the severity of depression, patients should receive integrated management components including optimization of cardiac treatment, psychosocial support, adherence reinforcement, and mental-cardiac bidirectional communication. Special subgroup assessment and follow-up are needed for post-ablation patients and elderly patients with frailty. The mediating and interactive effects of sleep quality, depression, and frailty in AF patients further support the importance of multidimensional assessment (95) (see **Figure 2** for details).

### 2.5 Future research directions

Based on existing research gaps and evidence strength, the following priority research areas are proposed, arranged by urgency and feasibility.

#### 2.5.1 High-priority unresolved issues

Human Mediation Analysis to Confirm Shared Mechanistic Pathways: Longitudinal cohort studies (prospective assessment of depression, collection of inflammatory, autonomic function, imaging, and electrophysiological parameters, follow-up for AF events) are needed, using structural equation modeling or causal mediation analysis to statistically verify the mediating effects of inflammation and autonomic dysfunction in the depression-AF relationship. This human causal inference is superior to animal models and extrapolation and represents a core breakthrough point in this field.

Randomized Controlled Trials of Psychological Interventions on Cardiovascular Endpoints: Currently, there are no clinical trials of psychological interventions with cardiovascular events (stroke, major bleeding, all-cause death, AF recurrence) as the primary endpoint. A multicenter, parallel-group randomized controlled trial is recommended, comparing usual care with usual care plus structured psychological intervention (e.g., CBT + behavioral activation) on cardiovascular outcomes in AF patients with comorbid depression.

Assessment of the Net Benefit of Antidepressant/Anxiolytic Medications on AF Outcomes: Current observational data suggest an inconsistent association between antidepressant use and AF risk, and antidepressants have not been shown to significantly alter AF risk in patients with heart failure with preserved ejection fraction (20, 94). Rigorous pharmacoepidemiological studies (e.g., using new-user designs, active comparators, propensity score matching) and randomized studies are needed to clarify the risk-benefit balance of pharmacotherapy.

Epidemiological and Intervention Studies in Low- and Middle-Income Countries: Existing epidemiological data and intervention studies are almost entirely from high-income countries, lacking systematic data on the prevalence, sociocultural determinants, and feasibility of interventions for AF with comorbid depression in low- and middle-income regions. Such studies are crucial for improving the global disease burden.

#### 2.5.2 Methodological recommendations

Standardized assessment tools: Future epidemiological and clinical studies should use validated, internationally recognized assessment tools (e.g., 12-lead ECG or continuous monitoring for AF diagnosis, MINI structured interview or PHQ-9 for depression assessment) to enhance comparability between studies.

Longitudinal Designs: Prospective cohort designs with adequate follow-up intervals (at least 2 years) should be prioritized to assess temporal dynamics and causality.

Quasi-Experimental Designs: When randomization is not possible (e.g., assessing the impact of depression on outcomes), quasi-experimental methods such as instrumental variable analysis, interrupted time series, and matched designs should be considered to reduce confounding bias.

Core outcome set: The field should develop a consensus-driven core outcome set for studies on AF with comorbid depression, including dimensions such as cardiovascular events, HRQoL, treatment adherence, depression improvement, and patient-reported experience.

#### 2.5.3 Translational research for drug target validation

For the P2X7R/NLRP3 pathway and IL-6 trans-signaling (see section 2.2.2 for details), mechanistic proof-of-concept studies in human AF patients should be conducted, including:

Obtaining atrial tissue specimens from patients undergoing cardiac surgery for depression-stratified molecular analysis;

Evaluating the effects of existing targeted drugs (e.g., IL-6 inhibitors are undergoing clinical trials for AF prevention) in the subgroup with comorbid depression;

Conducting immune-arrhythmia interventional trials needed to reclassify these pathways from “speculative” to “validated” or “refuted.”

The priority for future research is to validate mechanistic pathways proposed by animal models in human studies and to conduct adequately powered psychological intervention clinical trials with hard cardiovascular endpoints as the primary outcome. Methodologically, standardized assessment, longitudinal designs, and causal inference techniques should be elevated from prerequisites to routine practice. Filling the data gap in low- and middle-income countries is a prerequisite for improving global equity in care.

## 3 Conclusion

There is a complex, bidirectional pathophysiological and clinical relationship between AF and depression, which transcends the concept of “comorbidity” and constitutes a mutually reinforcing pathological cycle. From an epidemiological perspective, substantial evidence supports a consistent finding of an increased risk of AF associated with depression (with a modest but independent effect size) and a high prevalence of depression in AF patients (approximately 20–40%). From a mechanistic perspective, autonomic dysfunction and systemic inflammation are currently the most well-evidenced shared pathways, while novel pathways such as the P2X7R/NLRP3 inflammasome and IL-6 trans-signaling are primarily based on animal models, and their translatability to human disease requires validation in prospective studies. From a clinical management perspective, depression negatively impacts anticoagulation adherence, post-ablation recurrence, and overall quality of life, but the evidence from randomized controlled trials supporting specific intervention strategies remains relatively limited.

Integrating these multidimensional findings into clinical practice, we propose the following core tenets:

1. Routine screening for depression and anxiety in AF patients should not be considered optional but rather a basic standard of high-quality AF care.
2. Nurse-led comprehensive management combined with mental health support is currently the most feasible and evidence-supported intervention model for integrating AF and mental health care.
3. Cardiologists should be familiar with the basic interpretation of psychological assessment tools, identify patients requiring mental health referral, and understand how poor mental health affects cardiovascular prognosis through behavioral and biological pathways.
4. Future research must move beyond observational associations toward prospective interventional trials and human-based mechanistic validation to address the core bottleneck of weak translational evidence.

Ultimately, integrating mental health management into comprehensive AF care is not an added clinical burden but a strategic investment with the potential to improve the overall quality of AF management. By systematically bridging the gap between physical and psychological care, we can hope to improve the overall prognosis and quality of life for patients burdened by this dual disease.
